# Gel Electrolyte Constructing Zn (002) Deposition Crystal Plane Toward Highly Stable Zn Anode

**DOI:** 10.1002/advs.202104832

**Published:** 2022-01-19

**Authors:** Yu Hao, Doudou Feng, Lei Hou, Tianyu Li, Yucong Jiao, Peiyi Wu

**Affiliations:** ^1^ College of Chemistry Chemical Engineering and Biotechnology Donghua University Shanghai 201620 China; ^2^ Division of Energy Storage Dalian National Laboratory for Clean Energy Dalian Institute of Chemical Physics Chinese Academy of Sciences 457 Zhongshan Road Dalian 116023 China

**Keywords:** dendrite free, gel electrolyte, side reaction suppression, Zn (002) plane, Zn anode

## Abstract

Zinc (Zn) metal anode has been widely evaluated in aqueous Zn batteries. Nevertheless, the dendrite formation issue and consecutive side reactions severely impede the practical applications of Zn metal at high current densities. Herein, it is reported that engineering the gel electrolyte with multifunctional charged groups by incorporating a zwitterionic gel poly(3‐(1‐vinyl‐3‐imidazolio) propanesulfonate) (PVIPS) can effectively address the abovementioned issues. The charged groups of sulfonate and imidazole in the gel electrolyte can texture the Zn^2+^ nucleation and deposition plane to (002), which possesses a high activation energy to resist side reactions and induce uniform growth of Zn metal for a dendrite‐free structure. In addition, the Zn^2+^ solvation structure can be manipulated by the charged groups to further eliminate side reactions for high rate performance Zn batteries. Consequently, the polyzwitterionic gel electrolyte enables a stable cycling with a cumulative capacity of 3000 mA h cm^−2^ at high density of 7.5 mA cm^−2^ for the symmetrical Zn battery, and a long‐term cycling life for more than 1000 cycles at 5 C of Zn/MnO_2_ full battery. It is envisioned that the design of the gel electrolyte will provide promising feasibility on safe, flexible, and wearable energy storage devices.

## Graphical abstract

A polyzwitterionic gel electrolyte with charged groups of sulfonate and imidazole is proposed to regulate the Zn^2+^ solvation structure and texture Zn^2+^ deposition to (002) crystal plane simultaneously, which significantly resist the dendrite formation and side reactions, and dramatically boost the plating/stripping performance and long‐term cycling life even at high current densities.

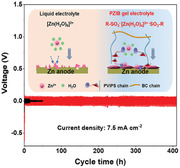

## 1 Introduction

**Figure 1:**
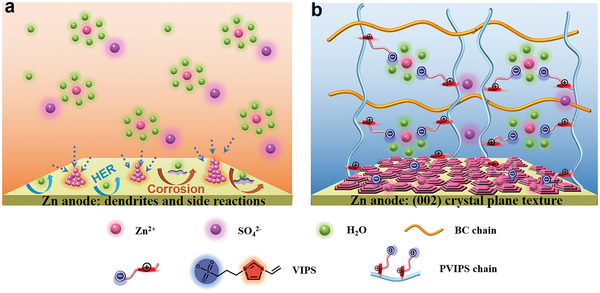
Texturing mechanism of the Zn anode with a) liquid electrolyte and b) PZIB gel electrolyte.

Aqueous Zn‐ion batteries with Zn metal as anode have been intensively investigated recently, owning to their promising potential on flexible and wearable batteries, such as high theoretical capacity, low cost, and high safety.^[^ ^1^, ^2^, ^3^, ^4^, ^5^, ^6^ ^]^ Nevertheless, the Zn anodes are suffering from the flagrant Zn dendrite and water‐induced side reaction issues which severely degenerate the long‐term cycling life‐span of Zn‐ion batteries especially at high current densities (**Figure** **1**a).^[^ ^7^, ^8^, ^9^, ^10^ ^]^ On the one hand, the uneven electric field distribution which is deteriorated at high current densities can induce the ion concentration and charge aggregation on Zn anode to facilitate the dendrite growth.^[^ ^11^, ^12^, ^13^, ^14^ ^]^ In addition, on account of the low surface energy, the primary (101) crystal plane is inclining to lead the Zn^2+^ deposition on dendrites.^[^ ^15^, ^16^, ^17^ ^]^ On the other hand, the Zn^2+^ solvation structure of Zn(H_2_O)_6_^2+^ can induce side reactions on Zn anode by triggering hydrogen evolution reaction (HER) and metal corrosions during cycling, which passivates the Zn anode surface, and renders inferior rate performance on plating/stripping.^[^ ^18^, ^19^, ^20^, ^21^ ^]^

To circumvent above obstacles, a great deal of strategies have been explored, including manipulating Zn deposition and nucleation behaviors to immunize dendrite formation,^[^ ^16^, ^22^ ^]^ regulating Zn^2+^ solvation structure for suppressing side reactions,^[^ ^23^, ^24^, ^25^, ^26^, ^27^, ^28^ ^]^ and modulating the surface electric field for uniform Zn deposition.^[^ ^11^, ^29^, ^30^ ^]^ In particular, texturing the (002) crystal plane during Zn^2+^ deposition has been demonstrated to be effective for dendrite and side reactions suppression recently.^[^ ^17^, ^21^, ^31^, ^32^ ^]^ As reported, the Zn (002) crystal plane owns high activation energy for Zn dissolution, which significantly resist the side reactions during cycling. Moreover, the typical Zn (101) and (110) crystal planes are mainly vertically aligned to the Zn surface and preferable for dendrite growth. In comparison, the hexagonal structure of the (002) crystal plane paralleling alignment to the Zn surface is more beneficial for Zn^2+^ uniform deposition to achieve the dendrite‐free anodes.^[^ ^33^ ^]^ Several strategies have already been demonstrated including graphene lattice driving^[^ ^28^, ^29^ ^]^ and sulfonate (SO_3_^−^) anions texturing for manipulating Zn (002) crystal plane^[^ ^21^ ^]^ so far. However, both the above design methodologies directly employ liquid electrolyte, which shows insufficient regulation on electric field distribution and Zn^2+^ solvation structure for Zn anode, and may still result in dendrite formation and side reactions at high current densities for long‐term cycling. Therefore, considerable designing a new strategy on (002) crystal plane construction is of great importance to facilitate reversible Zn metal anode.

Comparing to liquid electrolyte, gel electrolyte owns higher ion conductivity and safer performance on Zn‐ion batteries.^[^ ^2^, ^34^, ^35^, ^36^, ^37^ ^]^ Particularly, polyzwitterionic gel bearing anionic and cationic groups in the molecular chain can noticeably facilitate the ion migration for higher ionic conductivity and transference number.^[^ ^13^ ^]^ In addition, the sulfonate (SO_3_^−^) groups which can induce the Zn^2+^ for (002) homoepitaxial deposition are typical anions in many polyzwitterions.

Inspired by abovementioned results, here we proposed a design that employing the zwitterionic polymer PVIPS mechanically enhanced with bacterial cellulose (BC) as gel electrolyte (PZIB) on Zn‐ion batteries to modulate the Zn^2+^ coordination and manipulate the homoepitaxial deposition. In addition to proving high ionic conductivity, the charged groups (sulfonate and imidazole groups) in PVIPS can regulate the typical solvated structure [Zn(H_2_O)_6_]^2+^ to R‐SO_3_^−^ [Zn(H_2_O)_4_]^2+ −^SO_3_‐R, uniform the electric field distribution, construct and texture the Zn (002) crystal plane on Zn anode to significantly resist the dendrites and side reactions especially at high current densities (Figure 1b). As a consequence, the PZIB gel electrolyte enables the symmetrical Zn battery with superior cycling stability at high current density 7.5 mA cm^−2^ for over 400 h, and the Zn/MnO_2_ full battery with a reversible specific capacity of 150 mA h g^−1^ after 1000 cycles at 5 C.

## 2 Results and Discussion

### 2.1 PZIB Gel Electrolyte Design and Characterizations

**Figure 2:**
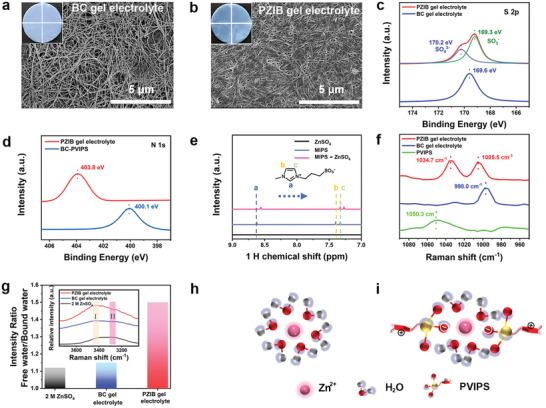
Structure characterizations of different electrolyte. Optical and field emission scanning electron microscopy (FESEM) images of a) bacterial cellulose (BC) and b) PZIB gel electrolyte. X‐ray photoelectron spectroscopy (XPS) spectra for different elements of c) S 2p, d) N 1s. e) Nuclear magnetic resonance (NMR) spectra of the 3‐(1‐methyl‐3‐imidazolio) propanesulfonate (MIPS), ZnSO_4_ and MIPS‐ZnSO_4_ regime. f) Raman spectra of different electrolytes and poly(3‐(1‐vinyl‐3‐imidazolio) propanesulfonate) (PVIPS) gel. g) Intensity ratio of free water and bound water in Raman spectra. Schematic illustration of the Zn^2+^ solvation structure in h) liquid electrolyte ([Zn(H_2_O)_6_]^2+^) and i) PZIB gel electrolyte (R‐SO_3_^−^ [Zn(H_2_O)_4_]^2+ −^SO_3_‐R).

The PZIB gel was prepared by polymerizing VIPS in BC network through the UV‐induced polymerization as depicted in Figure S1, Supporting Information. BC was pre‐soaked in DMSO solution to swell for aiding VIPS monomer infiltration.^[^ ^38^ ^]^ The obtained PZIB gel was immersed in liquid electrolyte to obtain the polyzwitterionic gel electrolyte (≈198 µm in thickness, Figure S2, Supporting Information), and no obvious swelling can be observed after storing in liquid electrolyte for 72 h (Figure S3, Supporting Information). **Figure** **2**a,b presents the optical and field emission scanning electron microscopy (FESEM) images of the translucence BC and PZIB gel electrolyte. Both BC and PZIB are porous structures, and the presence of PVIPS segments in BC provides a much denser 3D continuous network. The tensile stress–strain curves in Figure S4 (Supporting Information) indicate that the PZIB gel electrolyte owns an excellent mechanical strength of 7.1 MPa at the elongation of 62% which is much tougher than that of the PVIPS network.

In order to confirm the molecular interactions in the PZIB gel electrolyte, X‐ray photoelectron spectroscopy (XPS) and ^1^H nuclear magnetic resonance (NMR) have been employed. In Figure 2c, the spectrum of S2p for PZIB gel electrolyte consists of two peaks. Herein, the peak at 169.3 eV can be related to SO_3_^−^ of PVIPS, while the other peak at 170.2 eV can correspond to sulfur in SO_4_^2−^. As compared with that of pure ZnSO_4_ electrolyte, the S2p spectrum of SO_4_^2−^ presents a higher binding energy in PZIB gel electrolytes, indicating the variation of coordination environment of S element. Moreover, clear shift can be observed for the N1s spectrum of the imidazole ring after immersing the PZIB gel to 2 m ZnSO_4_ solution, confirming the variation of N element coordination environment (Figure 2d).^[^ ^39^ ^]^ Figure 2e further exhibits obvious shifts to higher field for the resonance peaks of imidazole protons H_a_, H_b_, and H_c_ in ZnSO_4_ solutions, revealing the electrostatic coupling of imidazole ring cations and SO_4_^2−^ anions.^[^ ^40^ ^]^ 3‐(1‐methyl‐3‐imidazolio) propanesulfonate (MIPS) which owns the same structure with PVIPS was employed here in the NMR characterization due to the poor solubility of PVIPS.

The solvation structure of Zn^2+^, which was altered in PZIB gel electrolyte, was then examined through Raman spectroscopy. As shown in Figure 2f, the band related to *v*‐ SO_4_^2−^ of ZnSO_4_ shifts from 998.0 cm^−1^ in BC gel electrolyte to 1005.5 cm^−1^ after the incorporation of PVIPS, indicating that the molecular interaction between Zn^2+^ and SO_4_^2−^ is interrupted with the presence of PVIPS. Meanwhile, the band of *v*‐SO_3_^−^ in PVIPS shifts from 1050.3 cm^−1^ to lower wavenumber of 1034.7 cm^−1^ in the PZIB gel electrolyte. From this point of view, it is inferred that in the PZIB gel electrolyte SO_3_^−^ groups in PVIPS could bond to Zn^2+^ through electrostatic interactions and consequently, change the solvation structure of ZnSO_4_. In the O−H stretching region of H_2_O, two bands located at 3420 (Figure 2g,I) and 3220 (Figure 2g,II) cm^−1^ related to free water and bound water, respectively, can be recognized.^[^ ^41^ ^]^ Higher intensity ratio of band I/II indicates larger amounts of free water molecules. Noticeably, the amount of free water will increase with more anions bonding to Zn^2+^. As shown in Figure 2g, the PZIB gel electrolyte presents the largest intensity ratio of band I/II, verifying that the amounts of water molecules coordinated with Zn^2+^ are distinctly reduced. The hypothesized mechanisms of the corresponding situations in liquid electrolyte and PZIB gel electrolyte are illustrated in Figure 2h,i, respectively. The typical solvation structure of [Zn(H_2_O)_6_]^2+^ in ZnSO_4_ liquid electrolyte is partially changed into R‐SO_3_^−^ [Zn(H_2_O)_4_]^2+ −^SO_3_‐R structures in PZIB gel electrolytes, which dramatically decreases the desolvation energy of Zn^2+^, thus facilitating the plating/stripping performance especially at high current densities.

### 2.2 Electrochemical Characterizations of the Zn Plating/Stripping Performance

**Figure 3:**
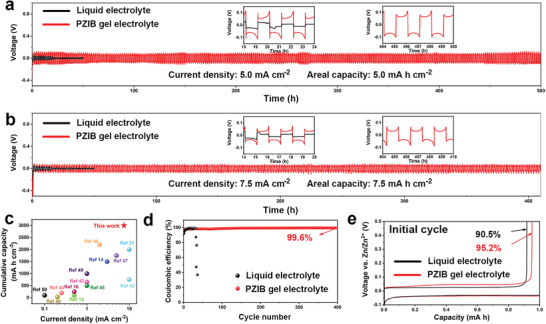
Electrochemical performances of the symmetrical and asymmetrical Zn batteries. Voltage profiles of the symmetrical Zn cells with liquid electrolyte and PZIB gel electrolyte at current density of a) 5  and b) 7.5 mA cm^−2^. c) Current density and cumulative capacity of the symmetrical Zn cells with PZIB gel electrolyte compared with current reported state‐of‐the‐art Zn‐ion batteries. d) Coulombic efficiency (CE) performances of the asymmetrical Zn/Cu cells with liquid electrolyte and PZIB gel electrolyte at current density of 1 mA cm^−2^. e) Voltage/capacity plots Zn/Cu cells with liquid electrolyte and PZIB gel electrolyte at the initial cycle.

Based on the analysis of the interactions among charged groups of polyzwitterions, Zn salt and the solvation structure of Zn^2+^, we deduce that with the present of polyzwitterions, the Zn^2+^ desolvation energy is much lower and the Zn plating/stripping performance at high current density can be improved. Accordingly, the Zn plating/stripping performance was evaluated by symmetrical Zn cells at different current densities. The performance of symmetrical Zn cells with different electrolyte at current density of 1 mA cm^−2^ is shown in Figure S5, Supporting Information. The battery with PZIB gel electrolyte can keep stable for over 1500 h, and the liquid electrolyte can only cycle for less than 100 h before wild fluctuation. Remarkably, at the high current density of 5 mA cm^−2^, and areal capacity of 5 mA h cm^−2^ in **Figure** **3**a, the PZIB gel electrolyte enhanced symmetrical Zn cell can cycle stable for more than 500 h, whereas the cell with liquid electrolyte can only cycle for less than 20 h before short circuit. In addition, even at the higher current density of 7.5 mA cm^−2^, and areal capacity of 7.5 mA h cm^−2^, the symmetrical Zn cell with PZIB gel electrolyte can still provide a sensational cyclic stability for over 400 h (Figure 3b), a ≈27‐fold longer than liquid electrolyte counterpart, which confirms the superiority of the PZIB gel electrolyte on dendrite suppression and side reaction inhibition. The cumulative capacity of symmetrical Zn cells with PZIB gel electrolyte can reach 3000 mA h cm^−2^ at the high current density of 7.5 mA cm^−2^, which surpasses most of current reported Zn batteries at high current densities as illustrated in Figure 3c.^[^ ^13^, ^14^, ^16^, ^21^, ^42^, ^43^, ^44^, ^45^, ^46^, ^47^, ^48^, ^49^, ^50^ ^]^ To further demonstrate that the advantages of PZIB gel electrolyte are mainly attributing to the polyzwitterions, BC gel electrolyte was also examined at different current densities as shown in Figure S6, Supporting Information. At 1 mA cm^−2^, the symmetrical battery can cycle for 400 h but with wide fluctuant. At high current density of 5 mA cm^−2^, the battery can only cycle stable for around 100 h, which are both much shorter than that of PZIB gel electrolyte.

The coulombic efficiency (CE) for plating/stripping procedure was examined by asymmetrical Zn/Cu cells. As presented in Figure 3d, the cell with PZIB gel electrolyte can deliver a CE of 99.6% after 400 cycles at the current density of 1 mA cm^−2^. In contrast, the liquid electrolyte asymmetrical cell can only operate for less than 30 cycles before a sudden decrease to less than 40% in several cycles. Furthermore, the initial CE which related to the side reaction levels of different electrolyte is also provided and compared here (Figure 3e), where the PZIB gel electrolyte is around 95.2%, higher than that of liquid electrolyte of 90.5%, demonstrating that the side reactions have already been suppressed from the initial stage in PZIB gel electrolyte..^[^ ^51^, ^52^, ^53^ ^]^

### 2.3 Mechanism Analysis for Dendrite Inhibition and (002) Crystal Plane Texture

**Figure 4:**
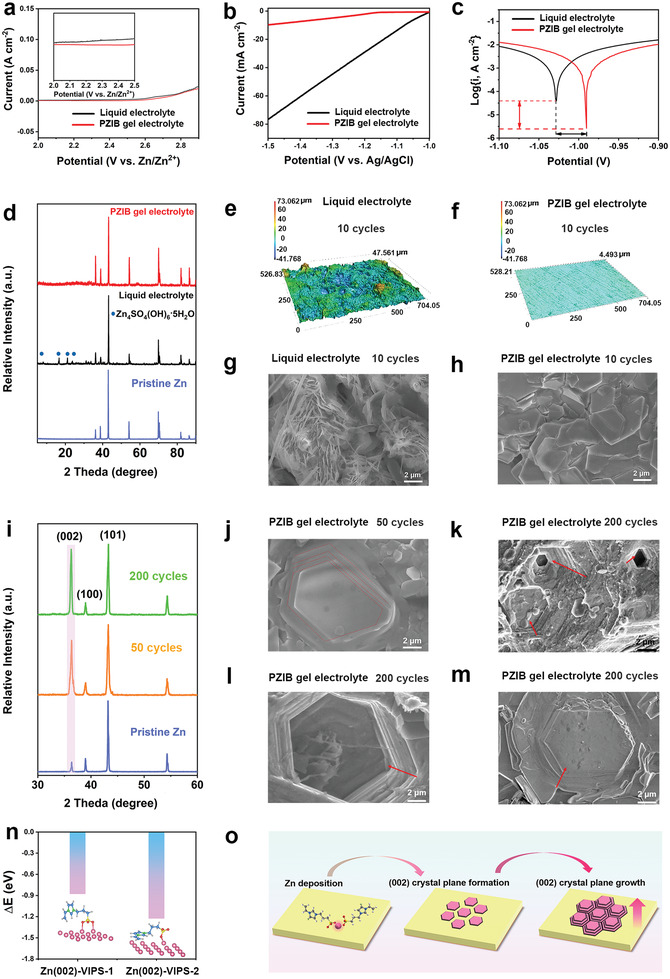
Side reaction and dendrite suppression mechanism of PZIB gel electrolyte. a) Electrochemical window of the PZIB gel electrolyte. b) Hydrogen evolution reaction (HER) curves and the c) Tafel tests for different electrolytes. d) X‐ray Diffraction (XRD) patterns of the pristine Zn, Zn anode after 10 cycles at 5 mA cm^−2^ in liquid electrolyte and PZIB gel electrolyte. Confocal laser microscopy (CLMS) images of Zn anode with e) liquid electrolyte and f) PZIB gel electrolyte after 10 cycles in symmetrical Zn cells, respectively. Field emission scanning electron microscopy (FESEM) images of Zn anode with g) liquid electrolyte and h) PZIB gel electrolyte after 10 cycles. i) XRD patterns of the symmetrical cells with PZIB gel electrolyte under different cycles at 5 mA cm^−2^. FESEM images of Zn anode with PZIB gel electrolyte after 50 j) and 200 k‐l) cycles in symmetrical Zn cells. m) FESEM image of Zn anode with PZIB gel electrolyte after 200 cycles, which was observed after storing for 6 months. n) Density functional theory (DFT) calculations for the adsorption energy of the VIPS to Zn (002) plane. o) Schematic illustration of the evolution of Zn (002) crystal plane during plating/stripping process.

To elaborate the failure mechanism of symmetrical Zn cells during plating/stripping, the electrochemical properties of different electrolyte were researched. As shown in **Figure** **4**a, PZIB gel electrolyte shows a broader electrochemical stability window of 2.5 V than that of the liquid electrolyte (≈2.0 V), demonstrating the electrochemical stability of PZIB gel electrolyte.^[^ ^15^ ^]^ Furthermore, the HER of different electrolytes were conducted in Figure 4b. Compared to the liquid electrolyte, the PZIB gel electrolyte presents a lower current response, revealing that the HER activity was suppressed in the PZIB gel electrolyte, which can be attributed to the optimized Zn^2+^ solvated structure.^[^ ^54^, ^55^ ^]^ Moreover, the corrosion behaviors of Zn anode in different electrolytes were evaluated by the Tafel tests (Figure 4c). As exhibited, the corrosion potential of Zn metal in PZIB gel electrolyte (−0.99 V) is higher than that of liquid electrolyte (−1.03 V), corresponding to the less corrosion tendency to Zn anode, which further verifies that the side reactions can be effectively mediated in PZIB gel electrolyte. Additionally, X‐ray diffraction (XRD) was employed to inspect the surface of pristine Zn metal, and Zn anode surface after 10 cycles with different electrolyte. As shown in Figure 4d, comparing to pristine Zn metal, the Zn anode in symmetrical Zn cells with liquid electrolyte shows obvious by product Zn_4_SO_4_(OH)_6_·5H_2_O peaks at 8.2°, 9.2°, 17.8°, and 19.2° after 10 cycles.^[^ ^15^, ^56^ ^]^ On the contrary, the XRD curve of Zn metal with PZIB gel electrolyte exhibits no obvious by product peaks after 10 cycles, which further confirms that the as‐designed polyzwitterionic structure can efficiently inhibit the formation of by products, so as to improve the Zn ion conductivity, Zn/Zn^2+^ reversibility of Zn batteries, and provide high‐rate performance. The three‐dimensional topography and surface detailed evolution morphology of Zn anode after 10 cycles with different electrolyte were obtained by confocal laser microscopy (CLMS) and FESEM, respectively. In Figure 4e,g, sharp peaks and protrusions are observed on the Zn anode surface with liquid electrolyte. Conversely, the Zn anode with PZIB gel electrolyte provides a dendrite‐free and dense surface (Figure 4f,h), which could be attributed to the efficient inhibition of side reactions. Optical images provided in Figure S7 (Supporting Information) further confirm that the surface of Zn anode with PZIB gel electrolyte after 10 cycles is much smoother than liquid electrolyte. Meanwhile, obvious sharp compounds (≈30.9 µm in thickness) can be observed in the Zn anode from the cross‐sectional images after 10 cycles with liquid electrolyte (Figure S8, Supporting Information). In stark contrast, the Zn anode keeps a smooth cross‐sectional surface with PZIB gel electrolyte. The electrochemical impedance spectroscopy (EIS) data further demonstrates that the PZIB gel electrolyte can effectively inhibit the dendrite and by‐product formation. After 10 cycles, the PZIB gel electrolyte battery renders a much smaller interfacial resistance change (from 204.8 to ≈245.0 Ω), comparing with liquid electrolyte (from 308.5 to ≈826.1 Ω) (Figure S9, Supporting Information). The XRD was also conducted to investigate the Zn metal crystal structure evolution during plating/stripping. As the ex situ XRD spectra indicated in Figure 4i, comparing to the diffraction peak of (101) crystal plane, the (002) peak increases significantly after plating/stripping at 5 mA cm^−2^ for 50 cycles. Moreover, with the increase of the cycling number at 5 mA cm^−2^, the (002) peak becomes stronger and stronger, demonstrating that the PZIB gel electrolyte can induce the Zn^2+^ to deposit along (002) plane (Figure S10, Supporting Information). The hexagonal (002) plane can also be unambiguously observed from FESEM images in Figure 4j–m. The homoepitaxial morphology has already formed after 50 cycles on Zn anode, with several layers of hexagonal Zn (002) aligned together and paralleling to the Zn metal surface (Figure 4j). It is noteworthy that, after 200 cycles at the ultrahigh current density of 5 mA cm^−2^ and areal capacity of 5 mA h cm^−2^, much more well‐defined and distinctly layered hexagonal Zn (002) structure stack together and parallel to the Zn metal substrate with no dendrite formation (Figure 4k,l). Even after storing for 6 months, the hexagonal Zn (002) structure can still be clearly observed (Figure 4m). In sharp contrast, the FESEM images of Zn anode with BC gel electrolyte in Figure S11 (Supporting Information) show a randomly flakes surface which is obviously distinct to that of PZIB gel electrolyte. Figure S12 (Supporting Information) displays that the N1s spectrum of PZIB gel electrolyte shifts to a lower binding energy after 50 cycles plating/stripping in symmetrical Zn cell, which could be ascribed to the enhanced interaction between the electrons on the Zn (002) crystal plane and the planar imidazole ring. Furthermore, density functional theory (DFT) calculations validate that with the planar imidazole ring paralleled along the Zn (002) plane and the SO_3_^−^ group stick to one Zn atom, the VIPS owns the stronger absorption energy (Figure 4n), illustrating that the SO_3_^−^ and imidazole ring in VIPS exhibit synergistic effect on the Zn (002) crystal plane depositing during plating/stripping. Therefore, we contribute these significant differences of the dendrite inhibition performance to the present of polyzwitterions, where the charged groups of SO_3_^−^ and imidazole ring can help to uniform electric field distribution, nucleation environment, and lead to a (002) plane homoepitaxial deposition as the conceptual diagrams illustrated in Figure 4o.

### 2.4 Electrochemical Performances of the Full Zn Batteries

**Figure 5:**
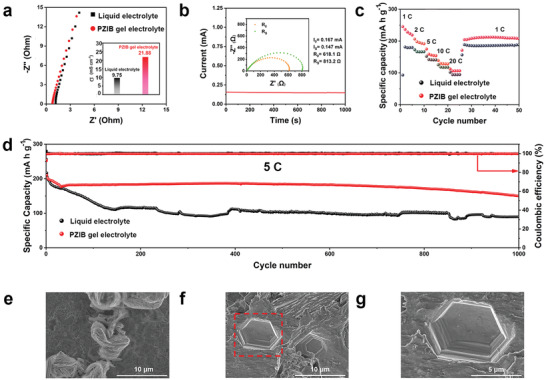
Electrochemical performances of the Zn/MnO_2_ full batteries. a) AC impedance curves of PZIB gel and liquid electrolyte, and the corresponding ionic conductivities in the inset image. b) Zn^2+^ transference number characterization of PZIB gel electrolyte. c) Rate performances of Zn/MnO_2_ battery at different current densities. d) Long‐term cycling performance of the Zn/MnO_2_ batteries with PZIB gel electrolyte and liquid electrolyte at 5 C. Field emission scanning electron microscopy (FESEM) images of Zn anode with e) liquid electrolyte and f–g) PZIB gel electrolyte after 1000 cycles in Zn/MnO_2_ batteries.

To further certificate the superiority of PZIB gel electrolyte on Zn‐ion batteries, ion conductivity and Zn^2+^ transference number are characterized. As shown in **Figure** **5**a, the ionic conductivity of PZIB gel electrolyte is calculated to be as high as 21.88 mS cm^−1^, which is much higher than liquid electrolyte (9.75 mS cm^−1^) and BC gel electrolyte (11.5 mS cm^−1^) (Figure S13, Supporting Information), attributing to the great Zn salt dissociation capability of polyzwitterions. Meanwhile, the PZIB gel electrolyte keeps a low electronic conductivity (4.20 × 10^−9^ S cm^−1^) as calculated in Figure S14, Supporting Information. The Zn^2+^ transference number is also evaluated by the symmetrical Zn cells and calculated to be as high as 0.74 from the chronoamperometry and impedance test, which is also higher than liquid electrolyte of 0.44 as exhibited in Figure 5b and Figure S15, Supporting Information. The high transference number can be ascribed to the less side reaction by‐products on the Zn anode surface, and the interactions between SO_3_^−^ groups and Zn^2+^, which decrease the Zn^2+^ transfer resistance on Zn anode, and improve the ion transfer path in gel electrolyte.

The Zn/MnO_2_ batteries were assembled and employed to manifest the cycling performance of full battery with PZIB gel electrolyte and liquid electrolyte. As tested, at the current densities of 1, 2, 5, 10, and 20 C (1 C = 308 mA g^−1^), the battery with PZIB gel electrolyte can provide a high reversible capacity of 230, 196, 159, 130, and 107 mA h g^−1^, respectively (Figure 5c), which are all higher than the performance in liquid electrolyte of 180, 167, 143, 119, and 95 mA h g^−1^, respectively. Long‐term cycling performance at high current density of 5 C is also evaluated for the batteries with PZIB gel electrolyte and liquid electrolyte in Figure 5d. As displayed, the PZIB gel electrolyte can deliver a high reversible specific capacity of 150 mA h g^−1^ for more than 1000 cycles, where the liquid electrolyte can only keep a capacity of 89.9 mA h g^−1^ after 1000 cycles. The corresponding characterizations of Zn/MnO_2_ batteries with BC gel electrolyte are also displayed in Figure S16, Supporting Information. As shown, the rate performances with BC gel electrolyte are 208, 180, 151, 125, and 100 mA h g^−1^, respectively, which are all lower than PZIB gel electrolyte (Figure S16a, Supporting Information). The long‐time cycling performance at 5 C is also unstable, providing a lower specific capacity of 101 mA h g^−1^ after 1000 cycles (Figure S16b, Supporting Information). Figure 5e presents the obvious Zn dendrite on the Zn anode surface after cycles in Zn/MnO_2_ battery with liquid electrolyte. By comparison, a dendrite free and clear hexagonal‐like structure is observed in the Zn anode using PZIB gel electrolyte, (Figure 5f,g), which guarantees the PZIB on inducing Zn homoepitaxial deposition for (002) plane growth. These results are all in line with the symmetrical Zn plating/stripping performances, and can also be attributed to the advantages of PZIB gel electrolyte on side reaction inhibition and Zn^2+^ deposition for (002) plane growth.

### 2.5 Flexible Batteries Applications

**Figure 6:**
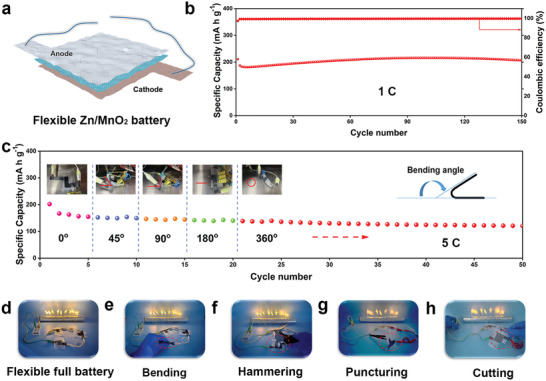
Electrochemical performances of the flexible Zn/PZIB gel electrolyte/MnO_2_ quasi‐solid state batteries. a) Schematic illustration of the full battery. b) Cycling performance of the flexible battery at current density of 1 C. c) Cycling performance of the flexible battery at different bending angle under the current density of 5 C. d) Digital image for the lights powering by flexible battery. e) Bending, f) hammering, g) puncturing, and h) cutting tests for the safety evaluation of the flexible batteries.

To demonstrate the practical applications on flexible and wearable devices, the Zn/PZIB gel electrolyte/MnO_2_ quasi‐solid state full batteries are assembled and characterized in open‐air (**Figure** **6**a). The long‐term cycling performance of the flexible battery is firstly examined at the current density of 1 C, as shown in Figure 6b. Attributed to the advantages of the PZIB gel electrolyte on Zn^2+^ transport and interfacial adhesion, the flexible battery can provide a high specific capacity as high as 206 mA h g^−1^ and keep stable for more than 150 cycles. Cyclic voltammetry (CV) curves of different cycles are shown in Figure S17 (Supporting Information) with the typical redox peaks of Zn/MnO_2_ battery. The 2^nd^ to 5^th^ curves were nearly overlapped, which convince the great cycling stability of the flexible batteries. The specific capacities under different bending angles are exhibited in Figure 6c at a high current density of 5 C. Under the bending angles of 0°, 45°, 90°, 180°, and 360°, the capacities of flexible battery are barely changed, which further prove the stability of the quasi‐solid state battery with PZIB gel electrolyte. When employed on practical applications, the flexible Zn/PZIB gel electrolyte/MnO_2_ full battery can easily power the lights as provided in Figure 6d. For further safety performance evaluations of the flexible batteries, bending, hammering, puncturing, and cutting tests were performed in Figure 6e–h. As expected, the batteries can still power the lights after above destructive experiments. All these results further indicate that the as‐designed PZIB gel electrolyte enhanced flexible batteries possess great potentials on serving as safe and wearable batteries.

## 3 Conclusion

In summary, we successfully demonstrated a gel electrolyte with multifunctional charged groups for effective Zn batteries side reaction inhibition and dendrite suppression. The large amounts of sulfonate and imidazole charged groups in the polyzwitterions play the role on regulating the solvation structure of Zn^2+^ to inhibit the side reactions on Zn anode. Moreover, the sulfonate and imidazole groups can uniform the electric field distribution and successfully texture the Zn^2+^ homoepitaxial to form the (002) plane, which further resist the dendrite formation and side reactions. Additionally, the charged groups can dedicate to the Zn salt dissociation ratio to improve Zn^2+^ transport, which result in the high ionic conductivity of 21.88 mS cm^−1^, and Zn^2+^ transference number of 0.74. Therefore, the Zn batteries with PZIB electrolyte provide excellent performance on plating/stripping and full battery cycling at high current densities. The flexible Zn batteries can provide the capacity as high as 120.6 mA h g^−1^ under current density of 5 C at different bending angles, and the safety performance is also confirmed by different destructive experiments. We anticipate that this work will shed light on constructing highly safe and dendrite free Zn batteries through polyzwitterionic gel electrolyte.

## 4 Experimental Section

### Materials

BC was purchased from Guilin Qihong Co., Ltd. DMSO was purchased from Shanghai Aladdin Biochemical Technology Co., Ltd. 1‐Vinylimidazole (97%), 1‐methylimidazole (99%), 2,2′‐azobis(2‐methylpropionamidine) dihydrochloride (V50), *N*,*N*’‐methylene bisacrylamide (MBA), Mn(CH_3_COO)_2_·4H_2_O, MnSO_4_, and KMnO_4_ were obtained from Sigma‐Aldrich Co., Ltd. W0S1009 carbon cloth was purchased from Ce‐Tech Co., Ltd. TGP‐H‐060 carbon paper was purchased from TORAY Co., Ltd. Carboxylated carbon nanotubes (CNT) were purchased from Suzhou Tanfeng Tech Co., Ltd. ZnSO_4_·7H_2_O, acetone, and Zn plates were provided by Sinopharm Chemical Reagent Co., Ltd. Zn foil was obtained from Qingyuan Metal Co., Ltd.

### Preparation of VIPS Monomer

A mixture of 1,3‐propane sulfonic acid (0.1 mol, 12.24 g) in 40 mL acetone was prepared and added into 60 mL acetone dissolved with 1‐vinyl imidazole (0.1 mol, 9.41 g) drop by drop at 0 °C in nitrogen atmosphere. Afterward, the mixture was stirred at room temperature for 3 days, following by filtration with acetone. The VIPS powder was then obtained by drying in a vacuum oven at room temperature.

### Preparation of BC Gel Electrolyte

A few pieces of BC pellicles (5 × 5 cm) are soaked into 2 m ZnSO_4_ and 0.1 m MnSO_4_ for 24 h at room temperature and then cut into regular circle (*d* = 19 mm).

### Preparation of PZIB Gel Electrolyte

VIPS (0.2 g), MBA (0.04 g), and V50 (0.01 g) were dissolved in 7 g water at room temperature and named as solution I. BC gel was soaked in DMSO for 30 min, and further infiltrated in solution I overnight before UV polymerization. The PZIB gel electrolyte was then obtained after washed several times with deionized water and further soaked in 2 m ZnSO_4_ and 0.1 m MnSO_4_.

### Preparation of the MnO_2_/CNT Cathode

The MnO_2_/CNT composite was prepared by a hydrothermal method according to previous literature.^[^ ^57^ ^]^ Typically, 0.15 g carboxylated carbon nanotubes and 2.94 g Mn(CH_3_COO)_2_·4H_2_O were dispersed in 150 mL water and stirred for 30 min before adding 1.27 g KMnO_4_. Afterwards, the mixed solution was transferred into the Teflon‐lined autoclave and heated for 12 h under 120 °C. The result mixture was centrifuged and washed with water for three times, and the MnO_2_/CNT composite was obtained after freeze‐drying. The MnO_2_/CNT cathode was prepared by coating CNT/MnO_2_, acetylene black, and PVDF mixture on carbon paper, and directly used after drying at 60 °C for 12 h.

### Preparation of Zn Anode

The Zn anode was prepared by cutting directly from pure Zn foil (≈27 µm in thickness) for semi‐cell assembly. For flexible battery, the Zn metal was prepared by electrodeposition at the current of 20 mA for 30 min through CHI 760D electrochemical workstation.

### Materials Characterization

XPS (Escalab 250Xi), Raman spectroscopy (Thermo Fisher Scientific DXR2xi), and NMR (Bruker AV‐400) were applied to study the interactions between PZIB gel electrolyte and Zn ions. XRD (Rigaku D/max‐2550VB+/PC) was employed to characterize the surface evolution of Zn anode. The morphologies of Zn anode and PZIB gel were observed by the FESEM (SU8230, Hitachi, Japan). The mechanical test was conducted on the universal testing machine (UTM2103, Shenzhen Sunstechnology Co., Ltd.).

### Electrochemical Measurements

Standard CR2016‐type coin cells were assembled for electrochemical performance assessment. All the cycling tests were performed on Neware battery testing system. Cyclic voltammogram (CV) was performed on CHI 760D at the scanning rate of 1 mV s^−1^ between the voltages of 0.8–1.85 V. Tafel test was conducted by the three‐electrode system with the Zn plate, platinum plate, and saturated calomel electrode (SCE) as working, counter, and reference electrode, respectively.

The ionic conductivity was characterized by EIS under the frequency range from 100 kHz to 1 Hz, and calculated as following equation:^[^ ^58^ ^]^

(1) $$\sigma =\frac{l}{R_{b}*S}$$

*l*: thickness of the separator; *R_b_*: bulk resistance; *S*: area of the separator.

The electronic conductivities of liquid electrolyte and PZIB gel electrolyte were measured by current‐time curves using symmetrical stainless steel (SS) cell.^[^ ^59^ ^]^ The electronic conductivities by the following Equation (2):

(2) $$\gamma =\frac{I_{ss}*L}{U*S}$$

where, *γ* is the electronic conductivity, *I*_ss_ is steady‐state current, *L* is the thickness of the electrolyte, *U* is the polarization voltage (1 V), *S* is the surface area of the electrolyte.

The transference number of Zn ions ($t_{Zn^{2+}}$) was calculated by testing the EIS from 100 kHz to 0.1 Hz with the symmetrical Zn cell before and after polarized under 10 mV for 1000 s. The calculating equation is as followed:^[^ ^60^ ^]^

(3) $$t_{Zn^{2+}}=\frac{I_{s}(\Delta V\;-I_{0}R_{0})}{I_{0}(\Delta V-I_{s}R_{s})}$$

Where, *I*_0_ and *R*_0_ are the initial current and initial resistance before polarization, respectively; *I*_s_ and *R*_s_ are the steady‐state current and steady‐state resistance after polarization, respectively; Δ*V* is the polarization voltage (10 mV).

### DFT Calculations

DFT calculations were carried out by Vienna Ab‐initio Simulation Package (VASP) with the projector‐augmented wave (PAW) method. All calculations were based on the same generalized gradient approximation (GGA) method. Perdew–Burke–Ernzerhof (PBE) functional was applied to deal with the exchange‐correlation term. Van der Waals interaction was taken into account at DFT‐D3 with Becke–Jonson (BJ) damping level. The plane wave cutoff was set to 500 eV. The Brillouin zone integration was carried out with 2 × 3 × 1 Gamma point for Zn‐VIPS. The convergence thresholds for energy and convergence thresholds for force were set as 10^−4^ eV during ion relaxation and 0.05 eV⋅Å^−1^, respectively. The absorption energy can be obtained by the following Equation (4),

(4) $$\Delta E=E\left(\mathrm{Surface}-\mathrm{absorbent}\right)-E\left(\mathrm{surface}\right)-E\left(\mathrm{absorbent}\right)$$

## Conflict of Interest

The authors declare no conflict of interest.

## Supporting information

Supporting Information

## Data Availability

The data that support the findings of this study are available from the corresponding author upon reasonable request.
