# Supplementary material for: Gel Electrolyte Constructing Zn (002) Deposition Crystal Plane Toward Highly Stable Zn Anode
Source: Adv Sci (Weinh). 2022 Jan 19;9(7):2104832. doi: 10.1002/advs.202104832 (PMC8895127; doi:10.1002/advs.202104832)
Supplement: Supplementary file 1 — Supporting Information [file ADVS-9-2104832-s001.pdf]

ADVANCED SCIENCE
Open Access

Supporting Information

for *Adv. Sci.,* DOI: 10.1002/advs.202104832

Gel Electrolyte Constructing Zn (002) Deposition Crystal Plane towards Highly Stable Zn Anode

Yu Hao, Doudou Feng, Lei Hou, Tianyu Li, Yucong Jiao, and Peiyi Wu**

Supporting Information

Gel Electrolyte Constructing Zn (002) Deposition Crystal Plane towards Highly Stable Zn Anode

Yu Hao, Doudou Feng, Lei Hou, Tianyu Li, Yucong Jiao, and Peiyi Wu**

This material includes

Materials and Methods

Figure S1 Synthesis of PZIB gel electrolyte.

Figure S2 The thickness of PZIB gel electrolyte.

Figure S3. Digital images of a) PZIB hydrogel and b) PZIB gel electrolyte.

Figure S4 Tensile stress-strain curves of different gel electrolyte.

Figure S5 Plating/stripping performances of symmetrical cells for PZIB gel electrolyte and liquid electrolyte at current density of 1 mA cm^{-2}.

Figure S6 Plating/stripping performances of symmetrical Zn cells for BC electrolyte at current density of 1 mA cm^{-2} and 5 mA cm^{-2}.

Figure S7 Optical images of Zn anode surface morphology for PZIB gel electrolyte and liquid electrolyte at 5 mA cm^{-2} after 10 cycles.

Figure S8. The cross-sectional images of the Zn anode after 10 cycles with different electrolytes at 5 mA cm^{-2}.

Figure S9. The EIS curves of the symmetrical Zn cells before and after 10 cycles with different electrolytes at 5 mA cm^{-2}.

Figure S10. The intensity ratio comparison of Zn(002)/Zn(101) after plating/stripping different cycles at 5 mA cm^{-2}.

Figure S11 FESEM images of Zn anode in symmetrical Zn cells with BC electrolyte after cycling for 50 cycles at current density of 5 mA cm^{-2}.

Figure S12 The N1s spectrum of PZIB gel electrolyte for initial and after 50 plating/stripping cycles.

Figure S13 Ionic conductivity characterization of BC electrolyte with AC impedance.

Figure S14. Current-time curves of symmetrical stainless steel (SS) cells with different electrolytes at a polarization voltage of 1 V at room temperature.

Figure S15 Zn^{2+} transference number characterization of liquid electrolyte.

Figure S16 Rate performance and long-term cycling performance of Zn/MnO_2 cell with BC gel electrolyte.

Figure S17 Cyclic voltammetry curves of the flexible battery at different cycles.

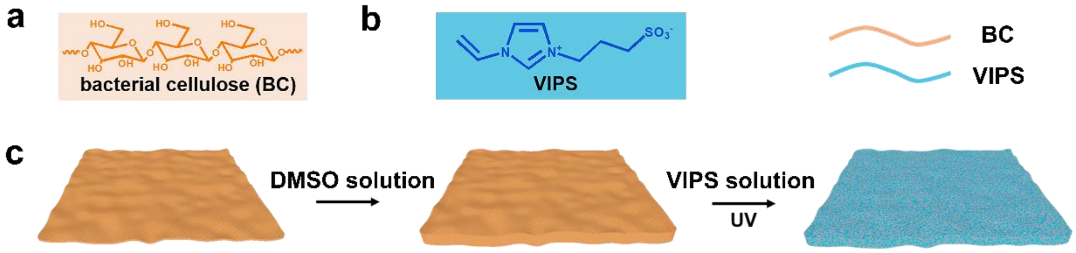

Figure S1. Synthesis of PZIB gel electrolyte.

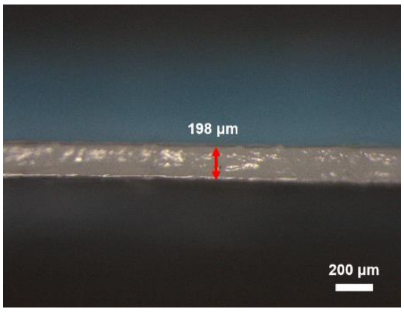

Figure S2 The thickness of PZIB gel electrolyte.

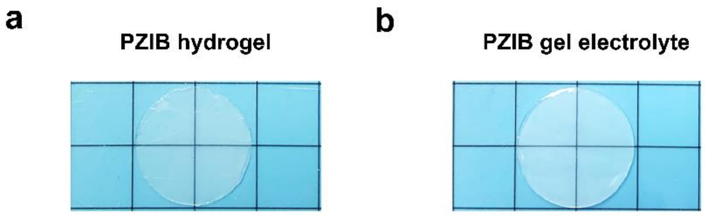

Figure S3. Digital images of a) PZIB hydrogel and b) PZIB gel electrolyte.

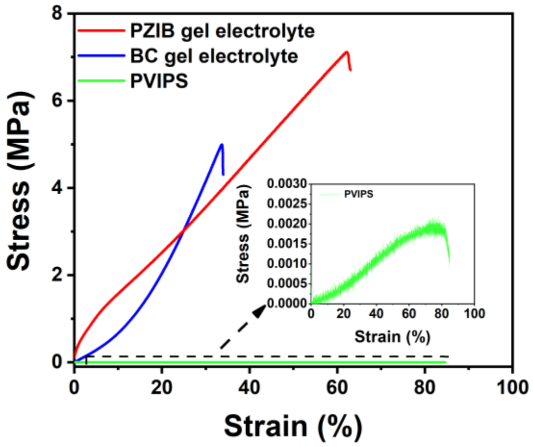

Figure S4. Tensile stress-strain curves of different gel electrolyte.

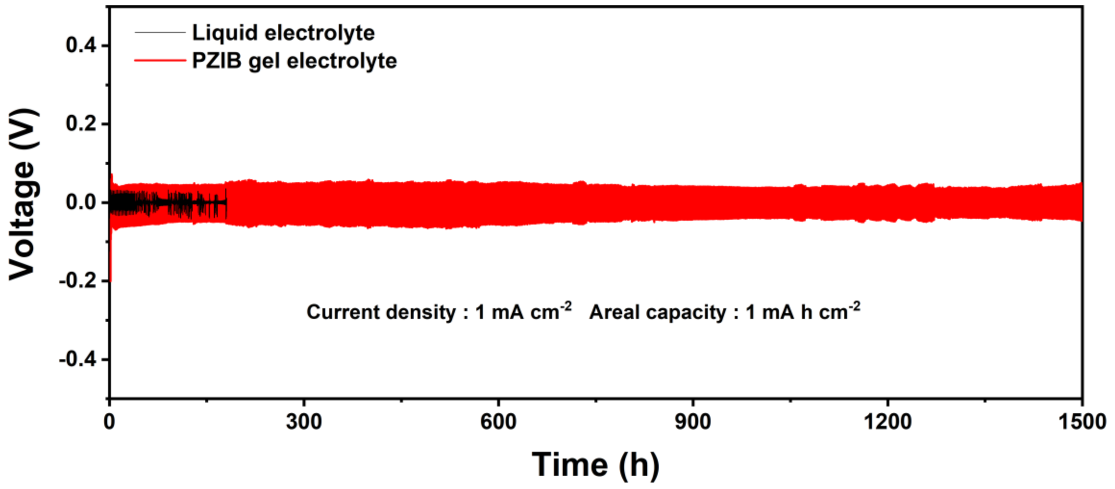

Figure S5. Plating/stripping performances of symmetrical cells for PZIB gel electrolyte and liquid electrolyte at current density of 1 mA cm^{-2} and areal capacity of 1 mA h cm^{-2}.

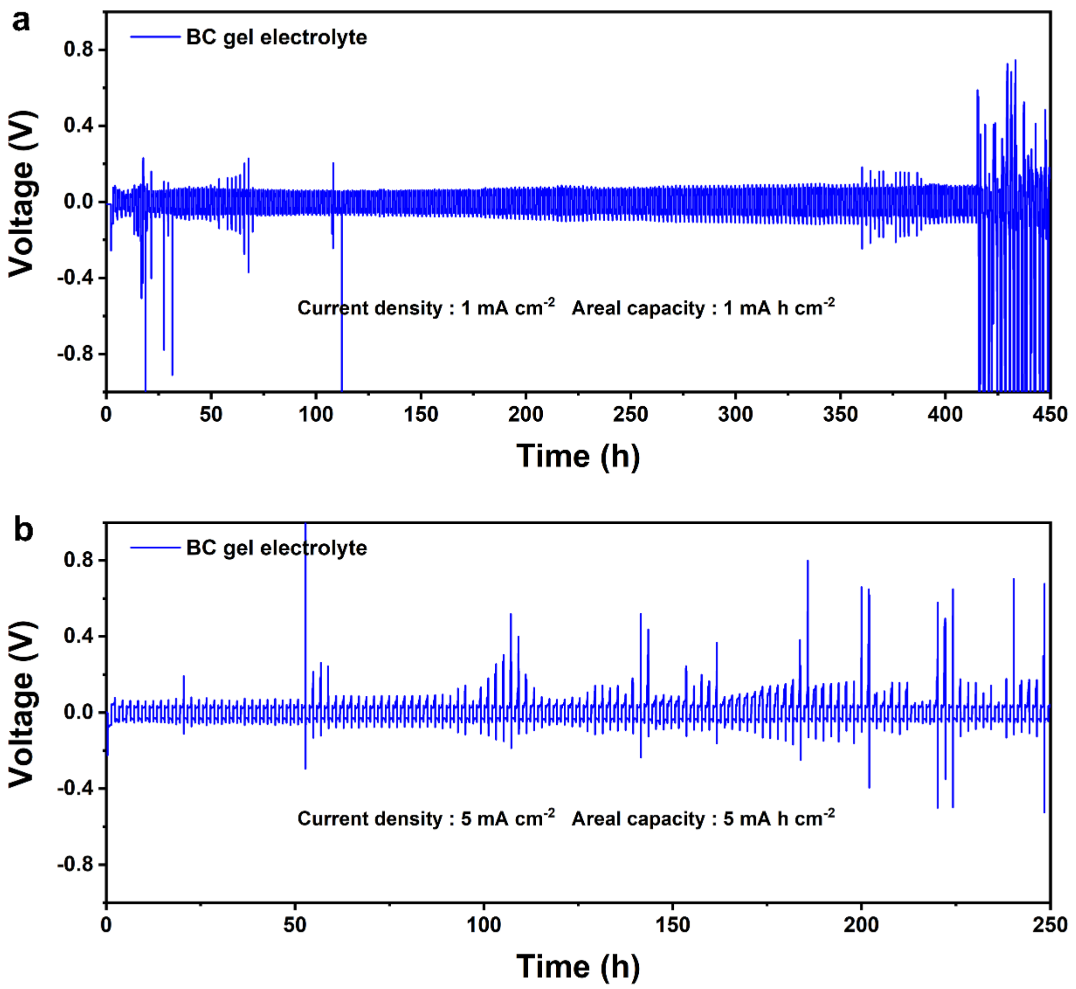

Figure S6. Plating/stripping performances of the symmetrical Zn cells for BC

electrolyte at current density of a) 1 mA cm^{-2} and b) 5 mA cm^{-2}.

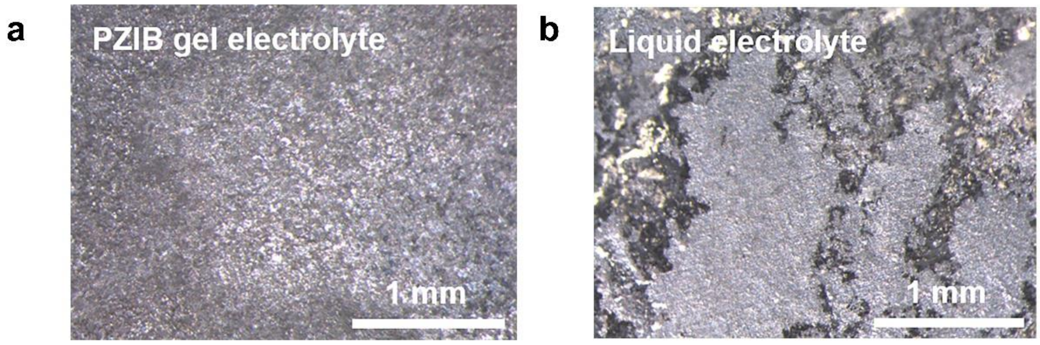

Figure S7. Optical images of Zn anode surface morphology for symmetrical Zn batteries with a) PZIB gel electrolyte and b) liquid electrolyte at the current density of 5 mA cm^{-2} after 10 cycles.

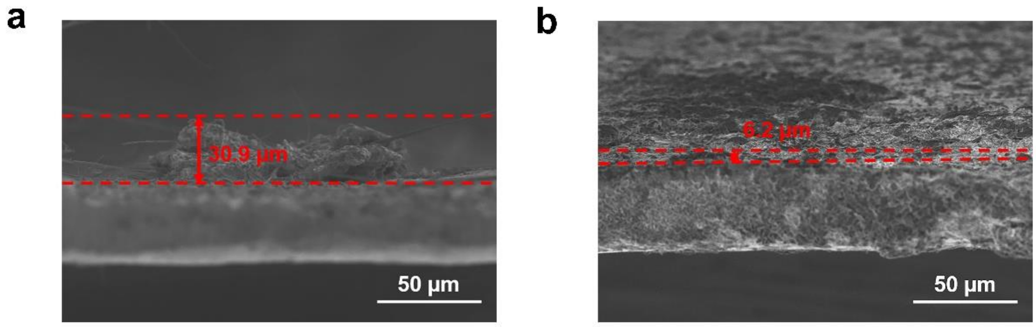

Figure S8. The cross-sectional images of the Zn anode after 10 cycles with a) liquid electrolyte and b) PZIB gel electrolyte at 5 mA cm^{-2}.

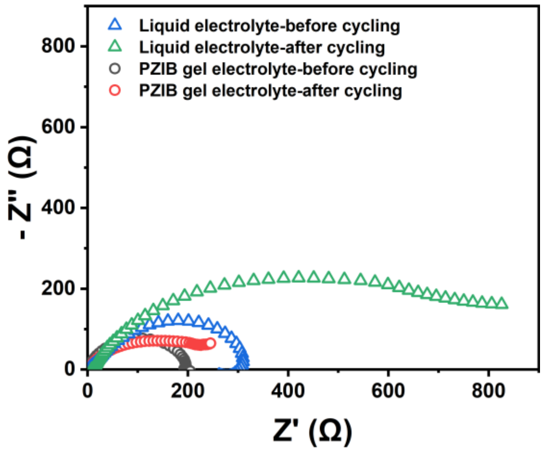

Figure S9. The EIS curves of the symmetrical Zn cells before and after 10 cycles at 5 mA cm^{-2}.

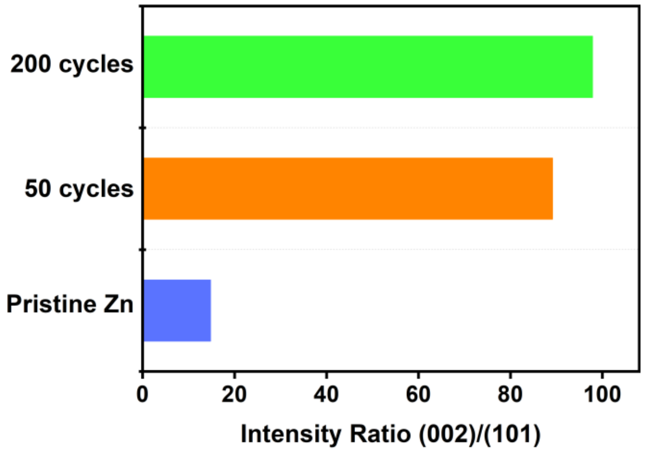

Figure S10. The intensity ratio comparison of Zn(002)/Zn(101) after plating/stripping different cycles at 5 mA cm^{-2}.

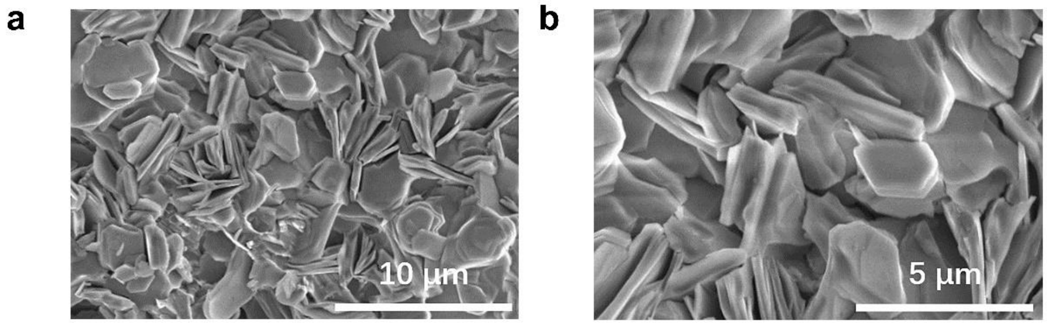

Figure S11. FESEM images of Zn anode in symmetrical Zn cells with BC electrolyte after cycling for 50 cycles at current density of 5 mA cm^{-2}.

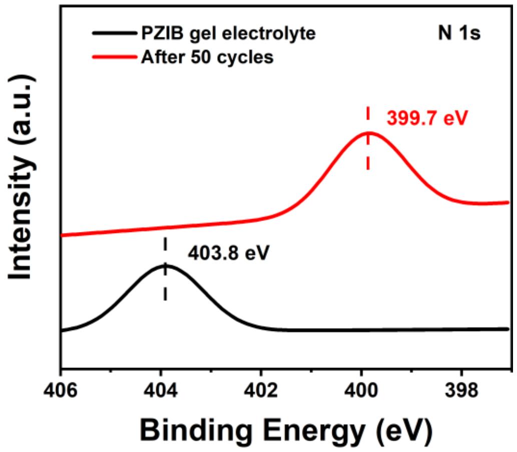

Figure S12. The N1s spectra of the PZIB gel electrolyte for initial and after 50 plating/stripping cycles.

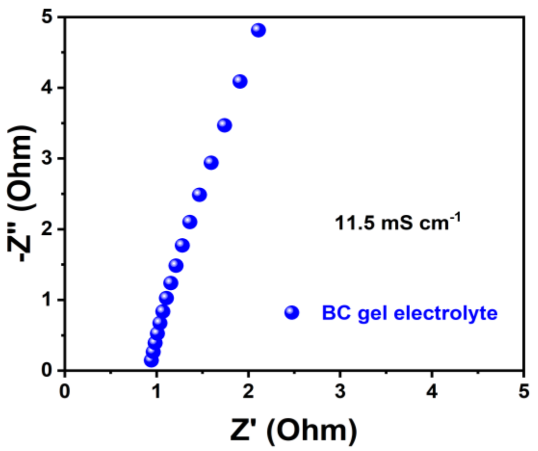

Figure S13. Ionic conductivity characterization of the BC electrolyte with AC impedance.

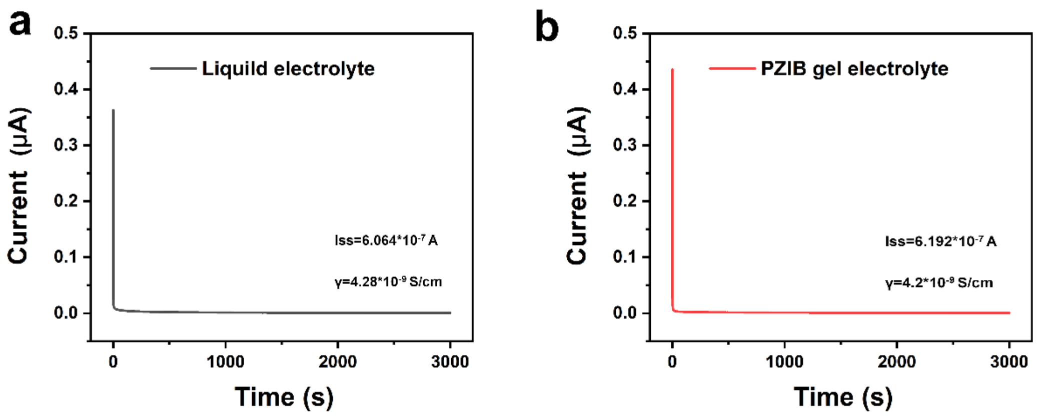

Figure S14. Current-time curves of symmetrical stainless steel (SS) cells with a) liquid electrolyte and b) PZIB gel electrolyte at a polarization voltage of 1 V at room temperature.

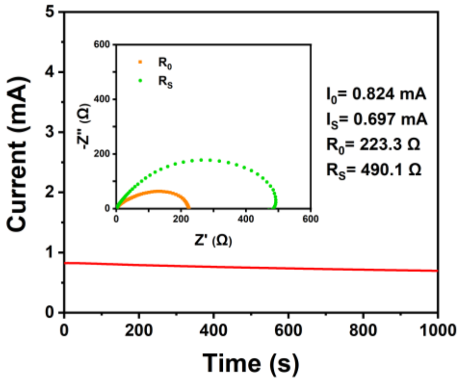

Figure S15. Zn^{2+} transference number characterization of liquid electrolyte.

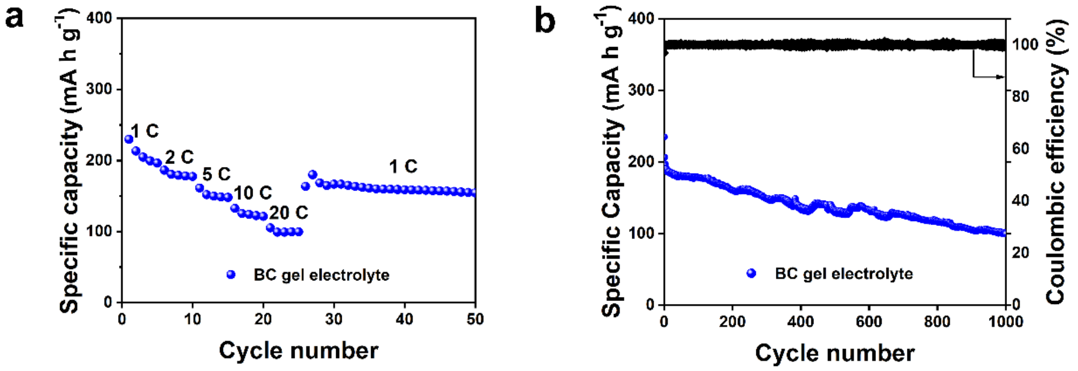

Figure S16. a) Rate performance of Zn/BC gel electrolyte/MnO_2 battery at different current densities. b) Long-term cycling performance of Zn/BC gel electrolyte/MnO_2 battery at the current density of 5 C.

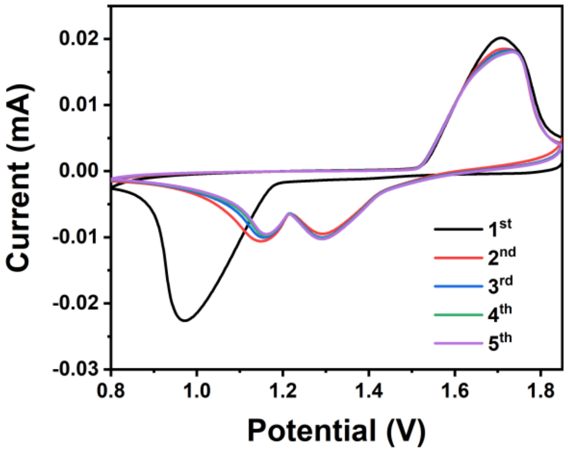

Figure S17. Cyclic voltammetry curves of the flexible battery at different cycles.
